# Croup—Quinine

**Published:** 1861-09

**Authors:** 


Dr. Eastman, Geneva, U. S., mentions the case of his daughter, about four years old, who was attacked with croup. The paroxysms were rather mild at first, assuming a periodical form, increasing in intensity from day to day, losing, as time advanced, more and more their paroxysmal form, taking on, gradually, the continued type or inflammatory character. All the ordinary remedies were early and effectually employed—as emetics, baths, mercurial purges, hot fomentations, blisters, etc., and were repeated as the case seemed to demand, the disease, meantime, progressing nevertheless, in spite of remedies, toward a fatal termination, in daily recur, ring, though less distinct, exacerbations and remissions, the fever assuming more and more a continued type. Losing all confidence in established practice, and reflecting on the effect of quinine in periodical diseases of every description, he resolved to try its effects in the case, confident that no harm could follow its administration as things were, and accordingly gave five grains, by weight, some three hours before the expected exacerbation, repeating the same quantity in one hour after. At the time anxiously looked for, as that of the approaching paroxysm, no signs of its return were manifested ; a cool sweat bedewed the child's face, the breathing remained soft and plsasant, and, in fine, no symptoms of the return of the croupy state appeared ; a calm, sweet sleep supervened, and the little patient awoke next morning every way better in appearance. The same course was pursued on the following afternoon, lest there should be a recurrence of the symptoms, with equally satisfactory results, after which, convalescence went on without interruption. In every succeeding case of croup, up to the present time, a repetition of this simple plan of medication, when it could be fairly put in practice and carried out, has been uniformly followed by results that have served to confirm his confidence in the course pursued; and he has not met with the first case, where this course was adopted in due time, that is, while any considerable exacerbations and remissions still remained, that did not completely yield to the treatment in question. Dr. E. does not hesitate to give ten or twelve grains of quinine to a child, one or two years old, within the space of two or three hours, and even more to children three and four years of age. He has never been able, by the most careful inquiries and observations, to discover that any headache or singing in the ears was experienced by the little patients; in fact, a complete tolerance of the medicine seems to exist in such cases.

When called during a paroxysm, he formerly employed emetics, and the other ordinary means usually resorted to in attacks of croup, to arrest the exciting symptoms; and, as these passed off, waited for further developments before resorting to the use of quinine. More recently he has ceased, as a general rule, to employ emetics, since they often render the stomach intolerant of the quinine, and thus prevent us from resorting successfully to the chief indication in the treatment, viz., the preventing of the return of the succeeding paroxysms, thus cutting short the disease; besides, the exhibition of a full dose of calomel, followed by the warm bath, continued a sufficient length of time—twenty-five or thirty minutes—produces, for the most part, all the relief that can be afforded by the most active emetic, without any of the unpleasant results so often following its use, as protracted nausea, and sometimes alarming prostration. When means are not at hand for the exhibition of the ordinary warm bath, enveloping the little patient in a warm or rather hot wet sheet, surrounded by a dry blanket to prevent too rapid evaporation, for an hour or two, will answer much the same purpose. *Ranking's Ab. No.* 31.

*Croup—Effect of some Remedies on the Membrane of.*—By Dr. Hermann, of St. Petersburg. The seventeen substances to be experimented with can be divided into four classes:

I. *Producing no perceivable alteration in the membrane:* Ether, absolute alcohol, tincture of iodine, solution of nitrate of silver, sesquichloride of iron, solution of caustic soda, and partly the solution of caustic potassa. The practical advantage derivable from some of these agents is less a change of the exudation, than their influence on the mucous membrane under it. Tincture of Iodine and sesquichloride of iron color the membrane, and the last named preparation also renders it more solid and tenacious. Nitrate of silver prevents the detachment of the membrane, by covering and compressing the same. A maceration of twenty-four hours in solution of caustic soda does not affect the membrane; but it appears shriveled in a solution of caustic potassa. The bichromate of potassa, as well as chromic acid, render the membrane friable, without alteration of its elements.

II. *Rapidly corroding agents:* The concentrated mineral acids. A few drops of nitric acid breaks up the membrane in a few seconds, it falling to pieces from the circumference toward the centre. The effect of muriatic acid is slower and less intense. Sulphuric acid converts the whole mass into coal. Acetic acid shows a very insignificant effect.

III. *Agents dissolving some of the constituents of the membrane.*—Chlorate of potassa rarefies the fibrin and renders the nuclei between it indistinct. Under the effect of a solution of ammonia, the nuclei and globules disappear, and the fibrin is broken up into moss-like groups. On the other hand, bromate and hydriodate of potassa dissolve the fibrin completely, leaving the globules and nuclei intact.

IV. *Complete dissolution of the membrane* is only effected by ammoniated copper. A few experiments with it on adults suffering from croup, seem to be encouraging enough for more extensive trials.

*Journal fur Kinderkrankheiten*, *Heft* 3 *u.* 4, 1860.

